# A non-invasive mouse model that recapitulates disuse-induced muscle atrophy in immobilized patients

**DOI:** 10.1038/s41598-023-49732-8

**Published:** 2023-12-14

**Authors:** Kun-Chang Wu, Hsiang-Wen Lin, Po-Chen Chu, Chia-Ing Li, Hsiang-Han Kao, Chih-Hsueh Lin, Yu-Jung Cheng

**Affiliations:** 1 School of Pharmacy, College of Pharmacy, China Medical University, Taichung, Taiwan; 2 Department of Cosmeceutics and Graduate Institute of Cosmeceutics, China Medical University, Taichung, Taiwan; 3 Department of Medical Research, China Medical University Hospital, Taichung, Taiwan; 4 Department of Family Medicine, China Medical University Hospital, Taichung, Taiwan; 5 Department of Geriatric Medicine, China Medical University Hospital, Taichung, Taiwan; 6 Department of Physical Therapy and Graduate Institute of Rehabilitation Science, China Medical University, Dr. Yu-Jung Cheng, No. 100, Section 1, Jingmao Road, Beitun District, Taichung City, 406040 Taiwan; 7 Department of Rehabilitation, China Medical University Hospital, Taichung, Taiwan

**Keywords:** Diseases, Medical research

## Abstract

Disuse muscle atrophy occurs consequent to prolonged limb immobility or bed rest, which represents an unmet medical need. As existing animal models of limb immobilization often cause skin erosion, edema, and other untoward effects, we here report an alternative method via thermoplastic immobilization of hindlimbs in mice. While significant decreases in the weight and fiber size were noted after 7 days of immobilization, no apparent skin erosion or edema was found. To shed light onto the molecular mechanism underlying this muscle wasting, we performed the next-generation sequencing analysis of gastrocnemius muscles from immobilized versus non-mobilized legs. Among a total of 55,487 genes analyzed, 787 genes were differentially expressed (> fourfold; 454 and 333 genes up- and down-regulated, respectively), which included genes associated with muscle tissue development, muscle system process, protein digestion and absorption, and inflammation-related signaling. From a clinical perspective, this model may help understand the molecular/cellular mechanism that drives muscle disuse and identify therapeutic strategies for this debilitating disease.

## Introduction

Immobility-induced muscle wasting (or disuse atrophy) represents a major health issue for patients who are bed-ridden for a prolonged period of time due to injuries or illnesses, which could incur further complications for the underlying conditions. Disuse atrophy remains an unmet medical need, in part, because the molecular and cellular mechanisms that drive the muscle wasting in the course of immobility remain unclear. To date, a number of animal models have been reported, including those of immobilization and mechanical unloading^1,2^. To simulate immobility atrophy, unilateral or bilateral immobilization of hindlimbs in rodents was developed^3,4^. Although these models have been widely used in the literature, several intrinsic problems warrant attention. For example, the cast immobilization method is commonly associated with skin injury, limb edema, and necrosis^5,6^, which become problematic in the context of animal welfare and data analysis. Although Velcro immobilization was developed to remedy these shortcomings^7^, this model, however, still caused severe skin erosion and bleeding, which led to premature termination of experiments in our hands.

In recent years, our research has focused on developing novel therapeutic strategies for different types of age- and disease-induced muscle atrophy, as exemplified by our recent identification of a Traditional Chinese Medicine (TCM)-based agent for the prevention of muscle wasting in a mouse model of cachexia^8^. However, this endeavor requires the access to different animal models, which allow rapid assessments of the in vivo anti-muscle atrophy efficacy of candidate therapeutic agents. Thus, in the present study, we report the development of a facile, non-invasive model of muscle disuse, which overcomes the aforementioned issues. Specifically, the use of preheated thermoplastic bandage allowed the fixation time, to a great extent, to be shortened, which could be easily reproduced in a lab setting. Moreover, use of the nontoxic polyvinyl siloxane as glue did not cause skin irritation or erosion. At the end of the 7-day experiment, this thermoplastic immobilization showed a significant reduction in the muscle weight and fiber size, reflecting mobility-induced muscle atrophy. The 12 weeks-old young mice which used in this study was to mimic the immobilization-induced atrophy in young patients after fracture or joints injury, whereas unloaded-atrophy is more often in elderly patients with bed-ridden.

From a translational perspective, this model recapitulates disuse atrophy in human patients, of which the utility is at least twofold. First, as thermoplastic mobilization dose not cause skin injury or limb edema, this model could help better understand the molecular mechanism that drives disuse atrophy via genomic analysis. Second, it could be used as an advantageous platform to identify therapeutic strategies that delay the onset of muscle wasting in the course of muscle disuse. Although RNAseq analysis on characterizing gene expression profiles in muscle atrophy has been reported in human and animal models^9–12^, we combined RNAseq followed by bioinformatics analysis to identify a series of genes associated with muscle tissue development, muscle system process, protein digestion and absorption, and inflammation-related signaling. The information here we provide could contribute more to disuse-induced muscle wasting. Moreover, this newly developed model which used unilateral immobilization-induced atrophy with lower inflammation provides an effective and efficient way for screening therapeutic drugs/methods for disuse atrophy.

## Materials and methods

### Animals

This animal study followed a protocol approved by the China Medical University (CMU) IACUC (CMUIACUC-2021-095). All experiments were performed in accordance with the Guideline for the Care and Use of Laboratory Animals of Taiwan Council of Agriculture Executive Yuan and the Animal Research: Reporting of in vivo Experiments (ARRIVE) guidelines. Twenty young male C57BL6 mice (eight-week-old, weight 24.1 ± 1.9 g) were purchased from National Laboratory Animal Center, Taiwan, and housed in the CMU Animal Facility with controlled room temperature (22 ± 1 °C), humidity (50 ± 10%), and lighting (12-h light/dark cycle). Mice were housed for another 4 weeks for environment adaptation. Later, mice were randomly divided into four groups, (1) negative control (n = 5); (2) immobilization for 7 days (Dis; n = 5); (3) immobilization for 7 days followed by rest for 7 days (Dis + 7D; n = 5); and (4) immobilization for 7 days followed by rest for 14 days (Dis + 14D; n = 5).

### Thermoplastic immobilization

**Figure 1:**
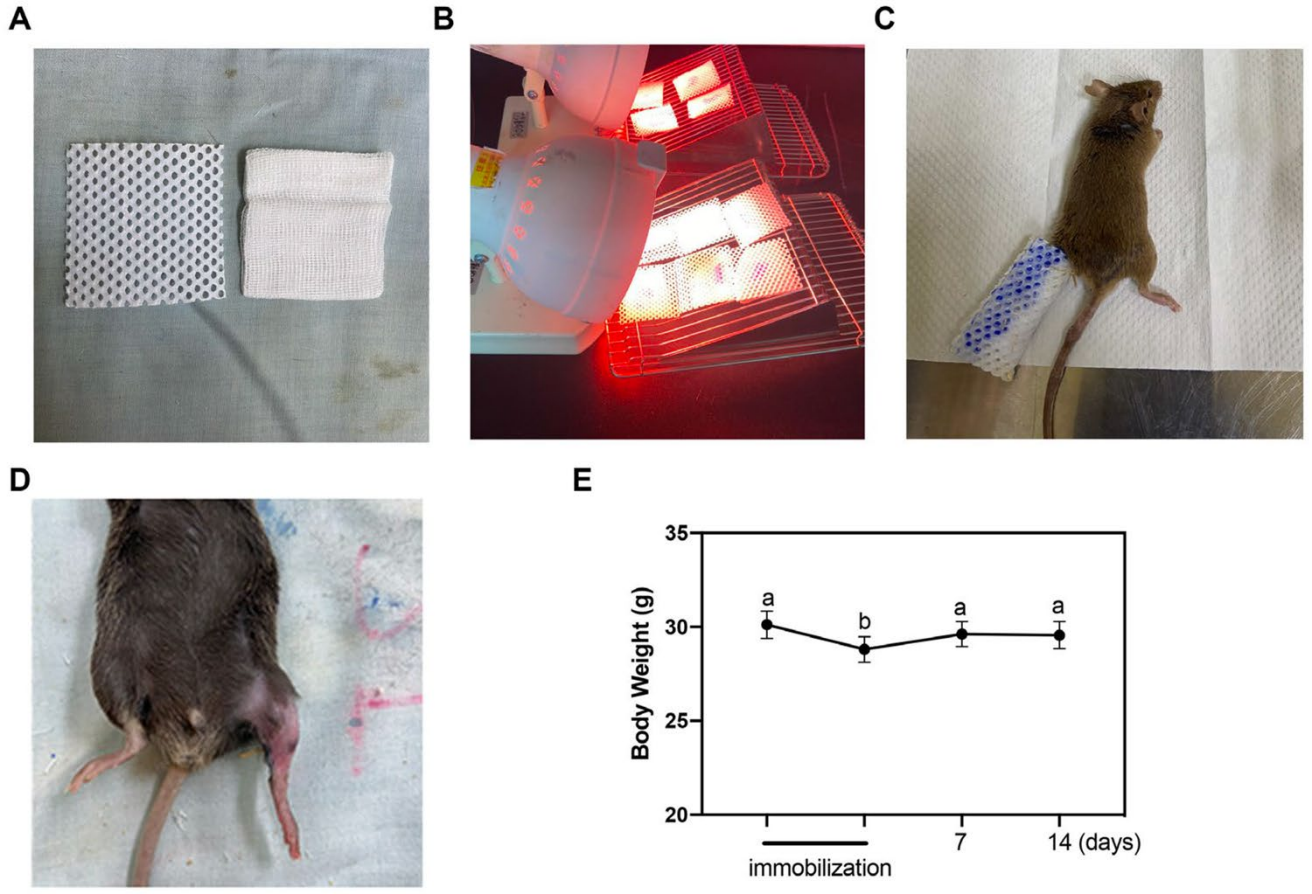
Thermoplastic bandage immobilization-induced disuse atrophy model. (**A**) Pre-cut thermoplastic bandage and gauze (size 2-inch × 2-inch). (**B**) Thermoplastic bandages were heated with infrared lamp. (**C**) Under anesthesia, polyvinyl siloxane was quickly applied on gauze/thermoplastic bandage to immobilize mice left hindlimb, with the hip and knee joints fixed at 180°. (**D**) Representative images of outlook of the immobilized hindlimb. (**E**) Statistical analyses were performed by repeated measurement one-way analysis of variance (ANOVA) followed by post hoc tests for body weight change during and after immobilization (n = 5). Different letters show statistical differences in the post hoc test when *p* < 0.05.

Thermoplastic bandage (Taipei Smart Materials Co., Ltd) was cut into 5 × 5 cm pieces, and heated under an infrared lamp to maintain softness and plasticity (Fig. 1A, B). The nontoxic impression material polyvinyl siloxane (Coltene AG, Alstatten, Switzerland) was use to glue gauze and thermoplastic bandage together. Mice were anesthetized under 3% isoflurane, and polyvinyl siloxane was quickly applied on gauze/thermoplastic bandage to immobilize the left hindlimb, with the hip and knee joints fixed at 180° (Fig. 1C). For avoiding escaping from bandage, mice were kept under anesthesia 20 min for polyvinyl siloxane hardening completely. The bandage was kept for one week, during which mice could move freely in the cage using the forelimbs and right hindlimb. These mice were monitored every day to check if the bandage became loose. At the end of the 7-day immobilization, mice were anesthetized under 2% isoflurane to remove thermoplastic bandage by cutting the adhesive edge.

### Tissue collections

At the end of experiments, mice were euthanized by cervical dislocation after anaesthetization under 3% isoflurane. Tibialis anterior (TA) and gastrocnemius (GC) muscles from both hindlimbs were isolated and weighed immediately after dissection, and were then frozen in liquid nitrogen, followed by − 80 °C storage, and fixed in 10% neutral buffered formalin (Leica Biosystems) respectively, which were later used for morphological and/or RNA-Sequencing (RNA-seq) analyses.

### RNA isolation

Total RNA was extracted using Trizol® Reagent (Invitrogen, USA) according to the instruction manual. Chloroform is used for phase separation of RNA (aqueous phase) from DNA (inter-/organic phase). Purified RNA was quantified at OD260nm using a ND-1000 spectrophotometer (Nanodrop Technology,USA) and qualitated by using a Bioanalyzer 2100 (Agilent Technology, USA) with RNA 6000 LabChip kit (Agilent Technology, USA).

### Library preparation, next-generation sequencing (NGS), and pathway analyses of differentially expressed genes (DEG)

RNA samples were prepared from the TA muscles of immobilized versus lateral non-mobilized legs by following the manufacturer’s protocol (Illumina, City, USA), and subsequent library constructions were conducted by using the SureSelect Strand-Specific RNA Library Preparation Kit (Agilent, City, USA), followed by size selection using AMPure XP beads (Beckman Coulter, City, USA). RNA-seq was carried out by a commercial vendor, Welgene Biotech (Taipei, Taiwan), via the Illumina's sequencing-by-synthesis technology. Sequencing data (FASTQ reads) were generated using the Welgene Biotech's proprietary pipeline based on the Illumina's basecalling program bcl2fastq v2.20. Both adaptor clipping and sequence quality trimming were performed using Trimmomatic (v0.36). HISAT2 program was used for mRNA alignment. Differential expression analysis was performed using StringTie (v2.1.3) and DEseq (v1.39.0). Gene ontology (GO) analysis, including the cellular component (CC), molecular function (MF), biological process (BP) and Kyoto Encyclopedia of Genes and Genomes (KEGG) pathway enrichment analysis, were carried out for DEGs using ClusterProfiler v3.6. Genes with low expression level (< 0.3 TPM value) in either or both of the treated and control samples were excluded. Genes with p value ≤ 0.05 and ≥ fourfold changes were considered significantly differentially expressed.

### Quantitative real-time polymerase chain reaction (qPCR)

One microgram of total RNA was used for the synthesis of complementary DNA, and qPCR was performed according to a procedure previously described^13^. Individual mouse primers used in this study were purchased from Thermo Fisher Scientific (Waltham, MA, S1). Normalization was performed by using GAPDH as the housekeeping gene, in which folds change in expression were calculated. Determinations were performed in triplicate for each gene of interest.

### Protein–protein interaction (PPI) network analysis

The STRING tool (https://string-db.org) database^14^ was used to delineate causal relationships of DEGs between control and disused muscle. The searching limitation set included text mining, experiments, databases, and co-expression, and “Mus musculus”. The interaction score > 0.4 was applied.

### Analysis of fiber cross-sectional area

GC, TA, and Soleus muscles were embedded in paraffin and sectioned at a thickness of 3 μm. Slides were subjected to immunohistochemical staining with anti-dystrophin antibody (ab275391, abcam) to perform qualitative fiber size measurements. Anti-MYH6 antibody (ab207926, abcam) was used to identify slow muscle fibers. Sections were examined and images were captured with a BX-43 microscope (Olympus America, Melville, NY) outfitted with a SAGE Vision SGHD-3.6C high-resolution digital camera (Sage Vision Co., Ltd, Taipei, Taiwan). ImageJ software (National Institutes of Health, Bethesda, MD) was used to perform quantitative measurements. All of individual muscle fibers were manually traced, and fiber areas of 180–250 muscle fibers were recorded in each slide.

### Statistics

All data were expressed as means with standard error for continuous variables. We examined the difference between two groups by using Mann Whitney test. A repeated measures ANOVA with Holm-Sidak test was used for body weight change. Analysis was conducted by using GraphPad Prism 8 software (GraphPad Software, La Jolla, CA, USA).

## Results

### Effects of thermoplastic immobilization on body weights, muscle weights, and fiber sizes

Throughout the 7-day period of immobilization, unilaterally immobilized mice remained physically active, and showed normal grooming and eating/drinking behaviors. Although hairs of immobilized hindlimbs were peeled off upon removal of the bandage, no obvious skin erosion or edema was noted (Fig. 1D). Figure 1E shows this unilateral immobilization caused a modest (5%), yet statistically significant, reduction in body weights relative to before immobilization [28.80 g versus 30.12 g; *p* < 0.05]. However, the body weight could be gradually recovered after the bandage was removed (29.62 g, 7 days post-removal).

**Figure 2:**
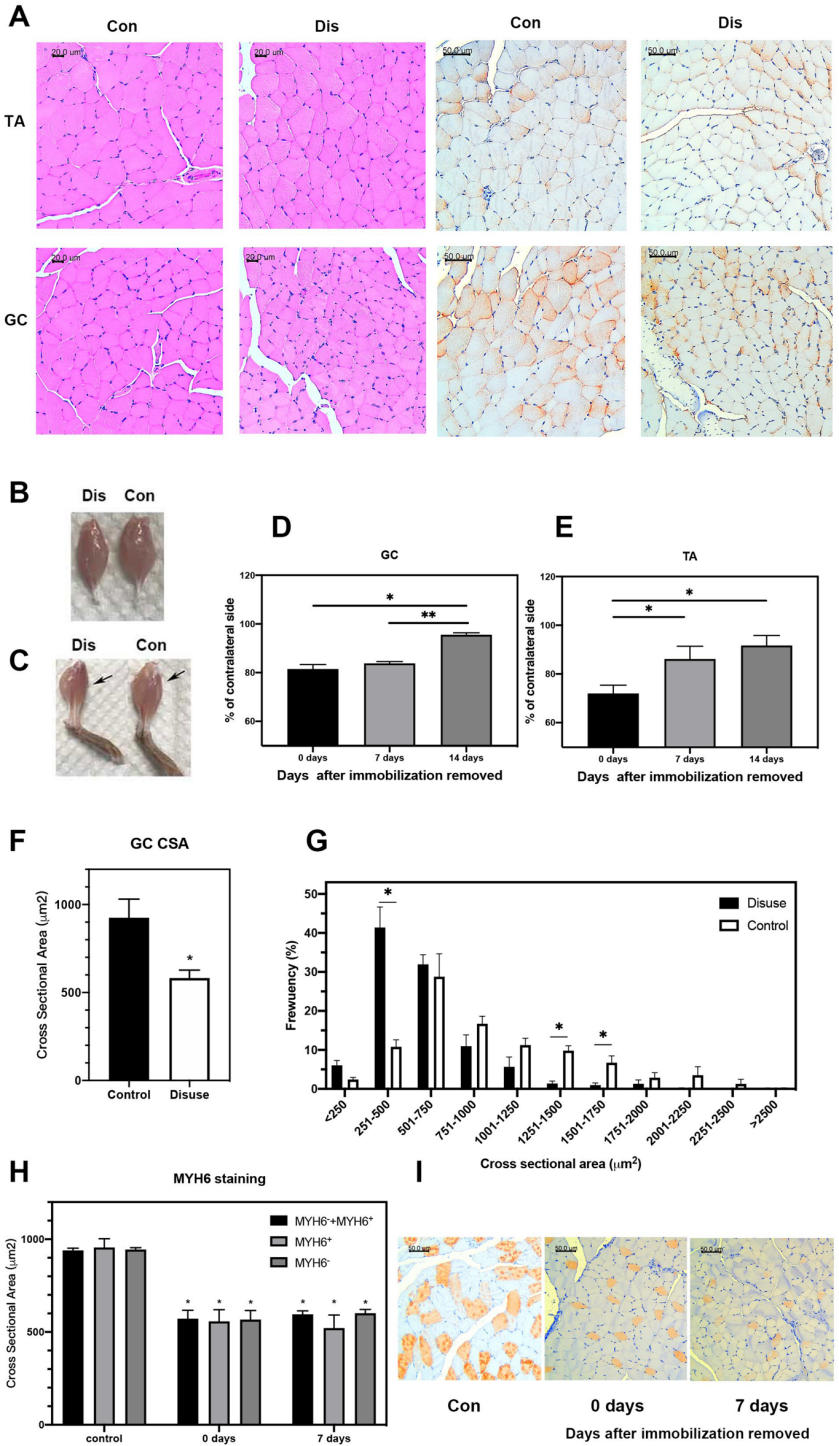
Outcome of Thermoplastic bandage immobilization immobilization. (**A**) Left: H&E staining of the TA and GC muscles. Scale bar = 20 μm. Right, Dystrophin immunostaining of the TA and GC muscles. Scale bar = 50 μm. (**B**) Representative pictures of the GC, and (**C**) TA muscles. The weight wet ratio of GC (**D**) and TA (**E**) of immobilization side to contralateral side at different time points. (**F**) Average cross-sectional area of GC muscle fibers. (**G**) Distribution of muscle fiber diameter of the GC muscle (n = 5). (**H**) Average cross-sectional area of MYH6+, MYH6−, or all muscle fibers of Soleus muscle. (**I**) MYH6 staining of the Soleus muscles (n = 3). The error bar denotes SEM. *,**represent p < 0.05 and p < 0.01, respectively.

Evidence suggests that this body weight loss was associated with immobility-induced muscle atrophy. First, a significant reduction in the GC and TA muscle of immobilized hindlimbs (DIS) was observed (Fig. 2B,C, representative images of muscles at the time of bandage removal). The GC weight at immobilized side relative to that of the contralateral, unimmobilized counterpart (CON), at different days were shown in Fig. 2D (bar graph representing % changes at days 0, 7, and 14 after bandage removal). The GC muscle weight of immobilized hindlimbs remained significant lower at Day 7 post-bandage removal (*p* < 0.01), but was able to return to that of the unconstrained counterpart at Day 14. TA muscles showed a similar trend in weight reduction but significantly improved at Day 7 and Day 14 post-bandage removal (Fig. 2E, P = 0.0278 and 0.0317).

Second, this muscle weight loss was accompanied by a parallel decrease in the muscle fiber size, as manifested by the H&E and anti-dystropin staining of the CG and TA muscles of immobilized (DIS) versus contralateral, unimmobilized hindlimbs (CON) at the end of 7-day immobilization (Fig. 2A, upper, TA; lower, CG). As shown, the mean cross-sectional area, per H&E staining, of the immobilized hindlimb of CG muscles was significantly smaller as compared to that of the contralateral counterpart (582.3 ± 101.9 μm^2^ versus 925. ± 236.8 μm^2^; *p* < 0.05, n = 5) (Fig. 2F). Furthermore, this reduced fiber size was accompanied by a shift of cross-sectional areas to the left, i.e., smaller sizes, in immobilized muscles (Fig. 2G). To identify if immobilization induces differential atrophy in slow/fast fiber types, we used myosin heavy chain 6 (MYH6), which expressed on slow skeletal muscle fiber, to explore the atrophy behavior on soleus muscle. As shown in Fig. 2H,I, the reduction of muscle fibers was observed in both MYH + and MYH- fibers, which indicated that the disuse-induced muscle atrophy may response similarly in both fast and slow fibers.

### Gene signature of skeletal muscle from mice with thermoplastic immobilization

**Figure 3:**
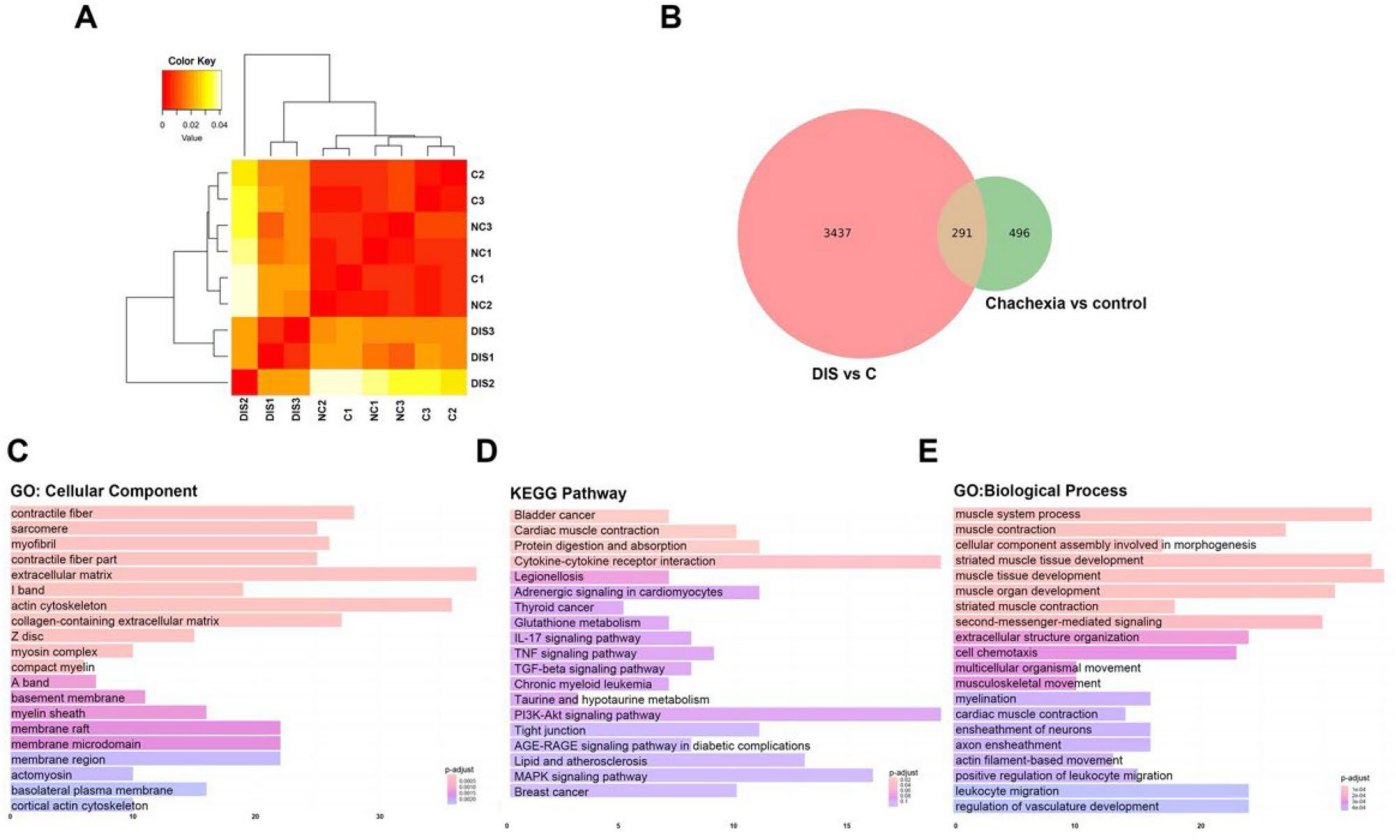
Gene signature of skeletal muscle from mice with thermoplastic immobilization. (**A**) Jensen-Shannon divergence in the topic distributions between different groups. (**B**) Venn diagram summarizing the overlap between differentially expressed genes from disused versus control (left circle) and cachexia versus control (right circle). (**C**) GO cellular component analysis, (**D**) KEGG pathways analysis, and (**E**) GO biological process analysis of the differentially expressed genes shared by the above two pairwise comparisons. GO, gene ontology; KEGG, Kyoto Encyclopedia of Genes and Genomes; Dis, disused; RNA‐seq, RNA sequencing; C, control; NC, negative control.

We performed transcriptome profiling on the gastrocnemius muscles isolated from three mice who had been immobilized with or without thermoplastic bandage for 7 days. We detected 17,597 expressed genes when counting genes with TMP over 0.1 in all 9 samples. Figure 3A shows that the gastrocnemius muscles samples from the negative control and the non-immobilized legs are similar and well separated from immobilized legs into distinct clusters in principal component analysis (PCA). We also analyzed sample diverge with single-sample-based Jensen-Shannon (JS) Divergence method. JS distance map also showed the gene expression profile from immobilized muscle is different to other two control groups. (Fig. 3A). When comparing the skeletal muscle gene signatures of the immobilized legs with CIM to the non-immobilized legs, we found 787 differentially expressed genes (p < 0.05 and fold change ≥ 4, supplementary data). Among these gene there were 454 genes were upregulated and 333 genes were downregulated. In addition, we compared gene profiles of immobilization-induced muscle atrophy with tumor-induced cachexia model, which provided in our previous study^8^ (Fig. 3B). Venn diagram analysis shows a total of 291 differentially expressed genes shared by the two muscle atrophy models (center portion) that showed changed expression pattern.

### Gene ontology (GO) enrichment analyses and KEGG pathways of differentially expressed genes

In order to identify key genes, gene ontology (GO) analysis including the cellular component (CC) and biological process (BP), and Kyoto Encyclopedia of Genes and Genomes (KEGG) pathway enrichment analysis 16 were accomplished.

Cellular component (CC) analysis obtained a total of 19 enrichment items; the targets were mainly enriched in extracellular matrix (38/549), actin cytoskeleton (36/549), contractile fiber (28/549), collagen-containing extracellular matrix (27/549), myofibril (26/549), sarcomere (25/549), and contractile fiber part (25/5492; Fig. 3C). Molecular function (MF) analysis obtained a total of 20 enrichment items, the targets were significantly involved in actin binding (28/535), receptor ligand activity (23/535), extracellular matrix structural constituent (18/535), tubulin binding (18/535), sulfur compound binding (16/535), and G protein-coupled receptor binding (16/535).

The most significantly cellular component enriched pathways associated with immobilization were “bladder cancer”, “cardiac muscle contraction “, “protein digestion and absorption “, “cytokine-cytokine receptor interaction “, “legionellosis, adrenergic signaling in cardiomyocytes “, “thyroid cancer “, “glutathione metabolism “, “IL-17 signaling pathway “, and “TNF signaling pathway “. Top twenty most overrepresented Kyoto Encyclopedia of Genes and Genomes (KEGG) pathways of differentially expressed genes are listed in Fig. 3D. Interestingly, among the 7 genes related to bladder cancer there are several genes associated with cell cycle, including Nras, Myc, Rps6ka5, Rb1, and E2f2. Differentially expressed genes from muscle tissues were mapped to 1270 different biological processes (BP), of which prominent examples are, response to muscle tissue development (35/543), striated muscle tissue development (34/543), muscle system process (34/543), muscle contraction (27/543) and second-messenger-mediated signaling (30/543; Fig. 3E). Tables 1 and 2 shows the 20 most significantly upregulated/downregulated genes and with largest absolute fold changes in response to immobilization (Disuse versus Control). Table 3 shows there is no significant expression difference in housekeeping genes.

**Table 1:** Genes up-regulated in the immobilized muscle.

Log2 ratio (Dis/Con)	NCBI Gene ID	Gene name	Gene description
17.35	20210	Saa3	Serum amyloid A 3
13.13	20311	Cxcl5	Chemokine (C-X-C motif) ligand 5
12.17	629970	Cd300ld2	CD300 molecule like family member D2
9.75	15267	H2ac18	H2A clustered histone 18
5.88	75697	C2cd4b	C2 calcium-dependent domain containing 4B
5.66	20208	Saa1	Serum amyloid A 1
4.14	51799	Rundc3a	RUN domain containing 3A
3.92	70729	Nos1ap	Nitric oxide synthase 1 (neuronal) adaptor protein
3.90	59083	Fetub	Fetuin beta
3.49	27428	Shroom3	Shroom family member 3
3.47	213043	Aox2	Aldehyde oxidase 2
3.22	211945	Plekhh1	pleckstrin homology domain containing, family H (with MyTH4 domain) member 1
3.18	15945	Cxcl10	Chemokine (C-X-C motif) ligand 10
2.91	12550	Cdh1	Cadherin 1
2.91	71522	Ggt6	Gamma-glutamyltransferase 6
2.79	64450	Gpr85	G protein-coupled receptor 85
2.68	13078	Cyp1b1	"cytochrome P450, family 1, subfamily b, polypeptide 1
2.57	434203	Slc28a1	Solute carrier family 28 (sodium-coupled nucleoside transporter), member 1
2.53	67888	Tmem100	Transmembrane protein 100
2.49	16477	Junb	jun B proto-oncogene

**Table 2:** Genes down-regulated in the immobilized muscle.

Log2 ratio (Dis/Con)	NCBI gene ID	Gene name	Gene description
− 13.09	20753	Sprr1a	Small proline-rich protein 1A
− 12.47	100039028	Mup11	Major urinary protein 11
− 11.63	380863	Tmem171	Transmembrane protein 171
− 5.60	67935	Ces5a	Carboxylesterase 5A
− 4.63	74338	Slc6a19	Solute carrier family 6 (neurotransmitter transporter), member 19
− 4.39	381835	Sbk3	SH3 domain binding kinase family, member 3
− 4.36	81799	C1qtnf3	C1q and tumor necrosis factor related protein 3
− 4.03	13009	Csrp3	Cysteine and glycine-rich protein 3
− 3.92	17883	Myh3	myosin, heavy polypeptide 3, skeletal muscle, embryonic
− 3.62	13190	Dct	Dopachrome tautomerase
− 3.58	68509	Ptx4	Pentraxin 4
− 3.45	240899	Lrrc52	leucine rich repeat containing 52
− 3.15	243078	Tecrl	Trans-2,3-enoyl-coa reductase-like
− 3.15	11464	Actc1	Actin, alpha, cardiac muscle 1
− 3.14	211949	Spsb4	splA/ryanodine receptor domain and SOCS box containing 4
− 3.14	216459	Myl6b	Myosin, light polypeptide 6B
− 3.04	170721	Papln	Papilin, proteoglycan-like sulfated glycoprotein
− 3.01	50706	Postn	Periostin, osteoblast specific factor
− 2.96	74488	Lrrc15	Leucine rich repeat containing 15
− 2.93	22402	Ccn4	Cellular communication network factor 4
− 2.85	71911	Bdh1	3-hydroxybutyrate dehydrogenase, type 1
− 2.82	104886	Rab15	RAB15, member RAS oncogene family

**Table 3:** Housekeeping genes in the immobilized muscle.

Log2 ratio (Dis/Con)	NCBI gene ID	Gene name	Gene description	p_value	q_value
1.3444	14433	Gapdh	Glyceraldehyde-3-phosphate dehydrogenase	0.2643	0.8641
0.3019	11461	Actb	Actin, beta	0.6134	0.9853
0.1011	12010	B2m	Beta-2 microglobulin	0.4936	0.954

**Figure 4:**
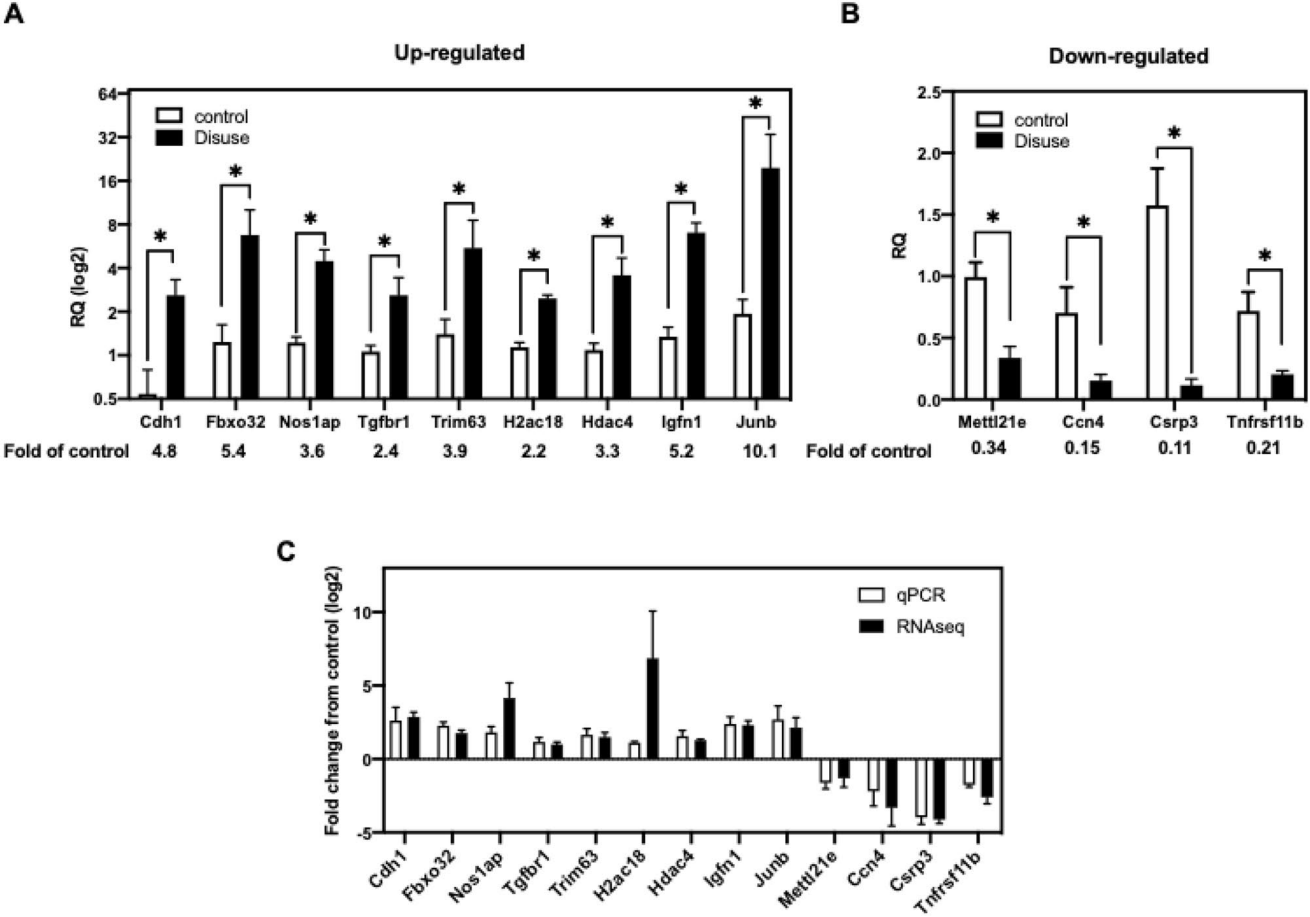
RT-qPCR analysis and relative fold changes between qPCR and RNA-seq (**A**) RT-qPCR analysis of nine up-regulated genes and (**B**) four down-regulated genes. (**C**) The comparison of RNA-seq and RT-qPCR fold change values obtained for 13 differentially expressed genes. Fold change values are represented in the log 2 scale and error bars represent SEM). P values by Mann Whitney test. *p < 0.05.

Nine up-regulated transcripts (Cdh1, Fbxo32, Nos1ap, Tgfbr1, Trim63, H2ac18, Hdac4, Igfn1, and Junb) and four down-regulated transcripts (Mettl21e, Ccn4, Csrp3, Tnfrsf11b) were selected for RT-qPCR analysis to prove the results of RNA-seq analysis (Fig. 4A,B). Moreover, the transcript expression fold-changes measured by RNA-seq and qPCR were high positive correlated, with a significant R2 value of 0.72 (Fig. 4C, p-value = 0.0033).

### Functional enrichment analysis of clusters

**Figure 5:**
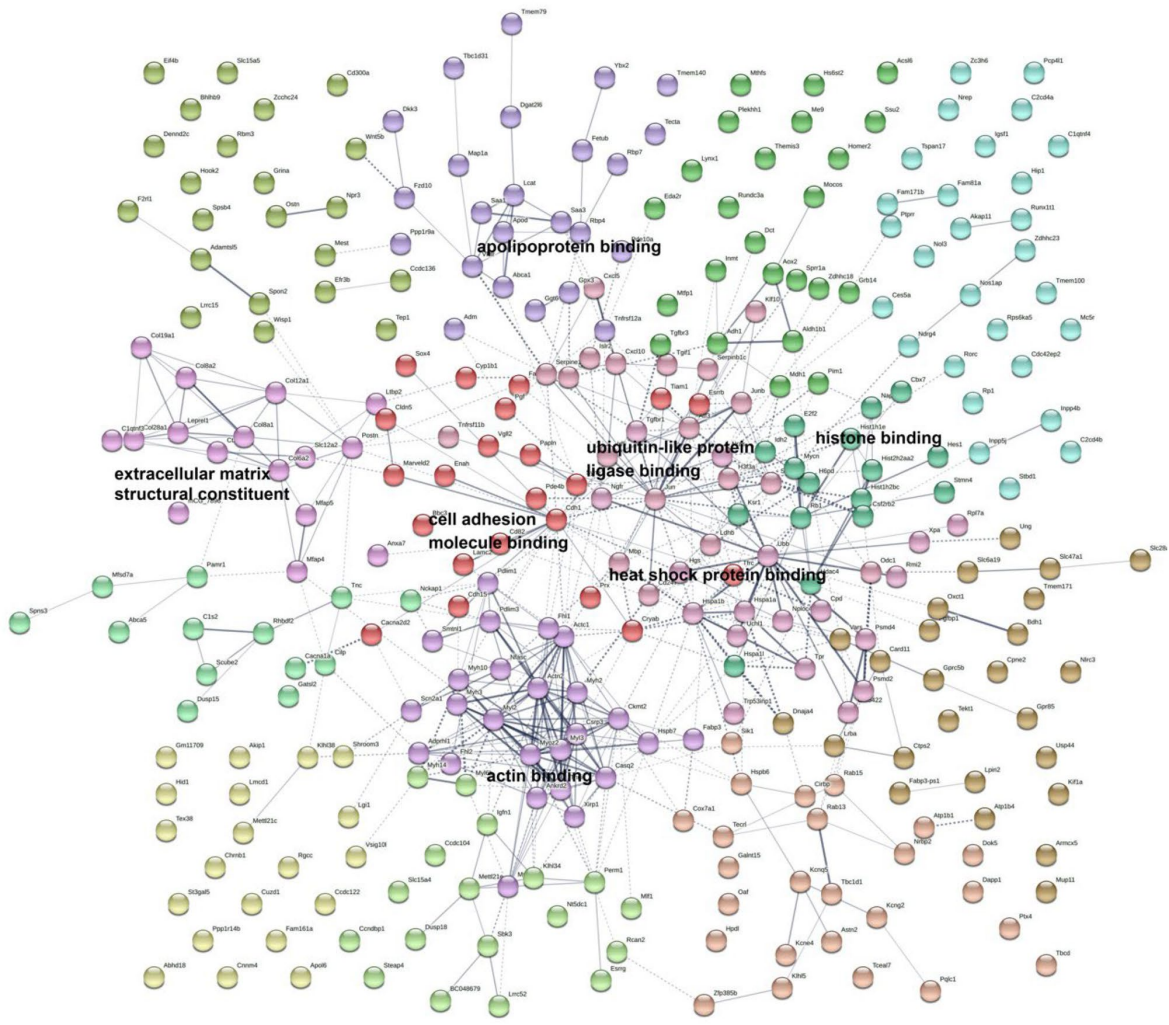
DEG PPI network analyzed using the STRING online database.

We further analyzed PPI of DEG using STRING. Genes with p value ≤ 0.05, q value ≤ 0.05, and ≥ twofold changes were included for PPI analysis. Figure 5 shows the 7 significant clusters that were found in the PPI network. In the cluster “extracellular matrix structural constituent”, the significant biological process and KEGG pathways were associated with “skeletal muscle organ development” and “protein digestion and absorption”. The genes included Col8a1, Col8a2, Col6a2, and Col19a1.

Cluster “cell adhesion molecule binding” was enriched in “cell–cell adhesion via plasma-membrane adhesion molecules” and “cell adhesion molecules”. Moreover, Enah in this cluster also involved in “Regulation of actin cytoskeleton”, whereas Pgf was correlated with PI3K-Akt and MAPK signaling pathways.

Several enriched biological processes and pathways in cluster “actin binding” were involved in muscle contraction, regulation of muscle contraction, and Apelin signaling pathway. For instance, myl2, myl3, myh3, myh10 and myoz2 were in this cluster. Genes in cluster “heat shock protein binding” were Hspa1a, Hspa1b, and Hspa1l. The biological process “response to heat” was enriched, however, no meaningful KEGG pathways were assigned to this cluster.

The biological functions of skeletal system development and response to oxidative stress was observed to be associated with cluster “ubiquitin-like protein ligase binding”. For example, Ngfr, Ubb, and Tgfbr1 genes were found in enriched in cluster “ubiquitin-like protein ligase binding”. Moreover, cluster “apolipoprotein binding” was observed to involved in biological functions of response to reactive oxygen species and lipoprotein metabolic process.

### Enrichment analyses of transcription factors

We further use transcription co-factors (TcoF) gene set enrichment tool (http://tcof.liclab.net/TcoFbase/Analysis/Analysis.php) to predict common upstream TcoF regulators. We input DEGs which used in 3.4 and separated these DEGs into up-regulated (156), down-regulated (168), and total groups (324). The candidate TcoFs were identified and listed in Table 4. There are more than 60 TcoFs were predicated using up-regulating DEGs. Among these, some TFs are reported be involved in regulating myogenesis or muscle fiber size, such as Ezh1, Cry1, Kdm4a, Crtc2…etc^15^. Interestingly, only 2 TcoFs were found might participate in down-regulating DEGs. Kmt2d and Med12 are associated with regulating muscle fiber size and muscle cell differentiation^16^.

**Table 4:** TcoF gene set enrichment.

TcoF	Annotated gene number	P-value
*Up-regulated DEGs*		
Ezh1	56	0.00011
Sumo1	67	0.00011
Cry1	60	0.00011
Kdm4a	61	0.00016
Hells	54	0.00018
Dpy30	57	0.00022
Crtc2	50	0.00023
Brdt	57	0.00024
Gmnn	60	0.00024
Tdg	58	0.00025
Per1	40	0.00026
Sirt6	56	0.00027
Cbx8	62	0.0003
Ell2	55	0.00031
Parp14	50	0.00031
Leo1	44	0.00034
Zmynd8	56	0.00037
Sf1	55	0.00038
Gtf2f1	56	0.00041
Smarcad1	39	0.00043
*Down-regulated DEGs*		
Kmt2d	77	0.00636
Med12	74	0.009
*Total DEGs*		
Kdm5c	119	0.0001
Hdac1	126	0.00011
Hdac3	125	0.00011
Ezh2	126	0.00012
Brd4	126	0.00012
Lmo2	120	0.00012
Trim28	124	0.00013
Kdm4c	124	0.00013
Auts2	92	0.00014
Atrx	125	0.00014
Smarca4	126	0.00014
Ep300	126	0.00014
Nipbl	121	0.00016
Kdm6a	120	0.00017
Per2	96	0.00018
Cdk9	121	0.00018
Baz1b	120	0.00019
Sirt1	121	0.00019
Cbx7	122	0.0002
Kdm1a	124	0.0002

## Discussion

Accelerating protein degradation and decreasing protein synthesis are two key events during muscle disuse which leading to muscle mass reduction^17,18^. Initiation of the ubiquitin-dependent proteolysis in skeletal muscle is the crucial trigger of muscle wasting^19^, and muscle atrophy F-box (MAFbx/atrogin-1) and muscle RING finger 1 (MuRF1), two E3 ubiquitin ligases, are frequently mentioned in different muscle atrophy models^20,21^. Insulin-like growth factor I (IGF1)/ phosphatidylinositol 3-kinase (PI3K), protein kinase B (AKT) and forkhead box O (FOXO) pathways, which are associated with anabolic pathways, regulate the expression of ubiquitin ligase^22^. Recently, anabolic medication, anti-inflammatory drugs, and enzymes inhibitors are considered as treatment options for muscle atrophy. Through clarifying genes involved in muscle atrophy, more effective treatments can be developed.

Many studies have shown the mRNA expression profile of muscle atrophy, mainly in denervation-induced and unloading-induced skeletal muscle atrophy^23–26^. In denervation-induced muscle atrophy, oxidative stress and inflammatory response genes are first up-regulated, whereas atrophy and atrophic fibrosis genes are induced later^24^. Similar to denervation-induced muscle atrophy model, the genes expression at early phase of unloading are related to stress response, including oxidative stress and cell proliferation inhibition. Proteolytic and inflammatory response genes are activated later^23^. Although the transcriptional profile at different time frames were reported in these two muscle atrophy models, only few studies investigate in immobilization-induced disuse atrophy. To find the precise information in immobilization-induced muscle atrophy, it is needed to conduct transcriptomics to analyze differentially expressed genes on suitable animal models.

In this study, we established new immobilization-atrophy mice model with minimal side effects and conducted RNAseq to analyze differentially expressed genes in mice gastrocnemius muscle at seven days after immobilization. Data were further analyzed by clustering and bioinformatic methods to figure out gene regulation in disuse skeletal muscles and might supplies potential targets for the prevention and treatment of weightlessness-induced muscle atrophy in the clinic.

The thermoplastic bandage immobilization created similar muscle atrophy in comparison with other immobilization method. As expected, the mice in our study demonstrated typical physiological responses to limb immobilization. That includes reduced muscle mass in hindlimb, decreased average CSA of muscle fibers, and frequency distribution of muscle fibers' CSA shifted toward to smaller size. Although thermoplastic bandage immobilization reduced body weight approximal 5%, no obvious adverse effects were noted. In order to illustrate the mechanism of immobilization-induced disuse atrophy, a gene expression profile was analyzed. Compared to the non-immobilized legs, a total of 787 DEGs were identified in muscle from immobilized legs, consisting of 454 up‐regulated and 333 down‐regulated genes. Compared with denervation method to study gene expression, thermoplastic bandage immobilization discovered several DEGs which showing the difference between these two disuse muscle atrophy types.

Among the top up-regulated and down-regulated DEGs, there are several genes were mentioned in previous studies. In addition to upregulated proteolytic genes such as MURF1 and ATROGIN-1, three genes control glucose homeostasis, NOS1ap, Inpp4b, and atf3, were also regulated in response to immobilized-induced muscle atrophy. The expression of NOS1ap, which encoded nitric oxide synthase 1 adaptor protein, was increased 3.6-fold in disused muscle than control. It was reported that overexpression of NOS1ap in obese mice can potentiated insulin-stimulated activation of IR/Akt in livers through its PDZ binding domain^27^. Inositol Polyphosphate-4-Phosphatase Type II (Inpp4b), which was down-regulated in disused muscle, can protect mice from high-fat diet metabolic dysfunction^28^. Atf3, which is also down-regulated in immobilized muscle, can preserve homeostasis in cardiomyocytes and controls peripheral glucose tolerance^29^. Atf3 overexpression can induce adipocyte browning and resistance to obesity in mice^30^. Moreover, C1qtnf3, which released by adipose tissue and regulates glucose homeostasis^31^, is significantly down-regulated in disuse muscle. These indicate that alteration of glucose/lipid metabolism plays an important role in immobilization-induced disuse muscle atrophy, which consisted with the infiltration of adipose tissue to muscle in patients with immobilization^32^.

Pieces of evidence show tumor necrosis factor and tumor necrosis factor receptor superfamily regulate muscle atrophy and regeneration. RANKL is connected to the progression of muscle atrophy, and RANKL inhibition can improve muscle strength and insulin sensitivity^33–35^. After RNAKL binds to RANK, downstream signals stimulate skeletal muscle atrophy. Tnfrsf11b, which encodes osteoprotegerin, can inhibit RANKL activity. In our results, gene expression of Tnfrsf11b is significantly reduced, while RANKL is upregulated in disuse muscle. Since the gene expression of RANK and downstream signal molecular, such as MAPKs and NF-kB, were not changed by immobilization, the disuse muscle atrophy might simply be induced by altering RANKL activity. Moreover, the expression of Tnfsf14, which encode LIGHT protein, was significantly increased by immobilization. Myocyte-derived Tnfsf14 is a survival factor necessary for myoblast differentiation and skeletal muscle regeneration^36^, thus, overexpression of Tnfsf14 might be due to inflammatory response initiated by immobilization. Another tumor necrosis factor receptor superfamily member Tnfrsf12a, also known as TWEAK receptor/Fn14, plays critical role in muscle atrophy. The up-regulation of Fn14 gene is reported in several muscle atrophy models such as denervation, immobilization, and hind limb suspension^37,38^. To our surprise, Tnfrsf12a is significantly down-regulated in disused muscle compared to control leg in our model. Compared to non-immobilized control leg, Tnfrsf12a gene expression is also lower in negative control mice, which from mice did not receive any immobilization. These results implied Fn14 overexpression may not present in all muscle atrophy conditions.

Growth hormone (GH) can improve muscle mass and GH-treatment has shown benefits muscle atrophy in denervation mice model^39^. Although GH itself was not changed by immobilization, there were some genes related to GH were regulated. Young and colleagues use GH knockout mice to evaluate the effects of GH treatment on muscle mass and related genes expression. Igfn1, which encodes immunoglobulin like and fibronectin type III domain containing 1, is predominantly expressed in skeletal muscle. Igfn1 shows it is required for myoblast fusion and differentiation. In our result, Igfb1 is up-regulated in disuse muscle while decreased in GH-treated mice^40^. Igfals, which encodes insulin-like growth factor binding protein acid labile subunit, is also induced in GH-treated model and was decreased to 0.69-fold in disuse muscle compared to control in our thermoplastic bandage immobilization. Moreover, insulin-like growth factor binding protein (Igfbp)-3, which was noted its low serum concentrations in atrophy muscle with poststroke patients^41^, is also downregulated in disused muscle. Similar patterns were also observed on Mettl21c, Sypl2, Hacd1, Cited4, Tceal7, and Klhl38 genes. These implied that immobilization-indued disuse atrophy might share similar pathway with GH-dependent muscle atrophy.

There are several limitations of our work. First, although positive correlations between mRNA and protein expression was found in several studies^42,43^, the increased mRNA not always leads to an increase in protein. We have examined protein level of MURF1 and ATROGIN-1, which were both increased in mRNA in disused groups. Although MURF-1 protein expression was increased in atrophy muscles, ATROGIN-1 protein was not changed significantly. Second, thermoplastic bandage method caused less skin irritation/edema but high escape rate if user is not skilled enough. The hardening time of polyvinyl siloxane depends activator concentration. In our preliminary trial, we found mice escaped from bandage in 10 min after woke up from anesthesia if polyvinyl siloxane was not dried completely. Thus, the suitable activator concentration and hardening time need to be tested for each user.

Thermoplastic bandage immobilization-induced hindlimb muscles disuse is correlated to inflammation responses and glucose/lipid metabolism, which resulted in striatum muscle atrophy. Notably, our model has less adverse effects and shows an easier to performed way to induce disuse muscle atrophy. This new approach may help understand the molecular mechanisms underlying immobilization-induced skeletal muscle atrophy and develop screening system to develop therapeutic drugs to prevent muscle loss on patients during immobilization.

### Supplementary Information

Supplementary Information 1.

Supplementary Information 2.

## Data Availability

The sequencing data regarding this study has been uploaded to the Gene Expression Omnibus (GEO) with Accession Number GSE237537 and is accessible via this link: https://www.ncbi.nlm.nih.gov/geo/query/acc.cgi?acc=GSE237537.
