# Supplementary material for: A non-invasive mouse model that recapitulates disuse-induced muscle atrophy in immobilized patients
Source: Sci Rep. 2023 Dec 14;13:22201. doi: 10.1038/s41598-023-49732-8 (PMC10721881; doi:10.1038/s41598-023-49732-8)
Supplement: Supplementary file 1 — Supplementary Information 1. [file 41598_2023_49732_MOESM1_ESM.docx]

Supplementary data:

Supplementary Table 1: List of gene names and log 2 ratio of Dis versus Control group

| **Gene name** | **log2ratio(DIS/Control)** |
| --- | --- |
| Sprr1a | -13.09384063 |
| Gm47328 | -13.00314601 |
| Mup11 | -12.47747327 |
| Gm34474 | -12.44072472 |
| Krtdap | -12.37173037 |
| Gm44717 | -11.96354517 |
| Gm13703 | -11.69579957 |
| Cryba4 | -11.64161677 |
| Tmem171 | -11.63286649 |
| Gm45140 | -9.266392517 |
| 2200002A13Rik | -8.555203004 |
| Gm45708 | -5.898900111 |
| Gm37336 | -5.85966916 |
| Gm39214 | -5.800267947 |
| Ces5a | -5.609688851 |
| H2ac10 | -4.821826886 |
| Gm49490 | -4.779422525 |
| Gm7870 | -4.732403726 |
| Slc6a19 | -4.633815013 |
| Sbk3 | -4.39482232 |
| C1qtnf3 | -4.366913737 |
| B930025P03Rik | -4.158258311 |
| Csrp3 | -4.033801991 |
| C130080G10Rik | -3.944746487 |
| Myh3 | -3.925446086 |
| 5430401H09Rik | -3.856129099 |
| Gm46637 | -3.717527777 |
| A730036I17Rik | -3.681911501 |
| Dct | -3.628195732 |
| Ptx4 | -3.58898316 |
| Gm20460 | -3.544037809 |
| Gm28653 | -3.51432891 |
| Gm33543 | -3.501756918 |
| Gm11757 | -3.462045497 |
| Lrrc52 | -3.45699808 |
| Gm8818 | -3.383789869 |
| H3c8 | -3.378472606 |
| Tecrl | -3.159341682 |
| Actc1 | -3.154961875 |
| Gm34455 | -3.154294143 |
| Spsb4 | -3.149832819 |
| Myl6b | -3.149201058 |
| Ripply1 | -3.07668326 |
| Pakap | -3.072592848 |
| Gm12295 | -3.069733275 |
| Papln | -3.04484833 |
| Postn | -3.019062963 |
| Lrrc15 | -2.96193313 |
| Ccn4 | -2.932297273 |
| 2610028H24Rik | -2.916257043 |
| Bdh1 | -2.85412458 |
| Rab15 | -2.821458689 |
| Gm31659 | -2.806884376 |
| Gm6212 | -2.689557799 |
| Gm41757 | -2.658445741 |
| Gm13490 | -2.655731762 |
| Rp1 | -2.655327906 |
| Tnfrsf11b | -2.651231705 |
| Ankrd2 | -2.64946074 |
| Strit1 | -2.649181573 |
| Tnc | -2.644110248 |
| Hectd2os | -2.636347251 |
| Hcar1 | -2.634531043 |
| Tnmd | -2.615252953 |
| Kera | -2.613933183 |
| Dok5 | -2.468493463 |
| Gm5532 | -2.433214127 |
| Col8a1 | -2.428859702 |
| Gm47708 | -2.364871247 |
| Aldh1a7 | -2.356887427 |
| Hspa1b | -2.336285341 |
| Kcng2 | -2.331760651 |
| Gm32391 | -2.32868526 |
| Col12a1 | -2.319577898 |
| Wnt16 | -2.283955197 |
| Hspa1a | -2.278529414 |
| Gm19410 | -2.273280393 |
| Agrp | -2.270378134 |
| Nlrc3 | -2.236843563 |
| Ltbp2 | -2.221045123 |
| Casq2 | -2.210886558 |
| Ckmt2 | -2.203171242 |
| Myl2 | -2.187673629 |
| Tnni1 | -2.180287343 |
| Gm15543 | -2.160915541 |
| P3h2 | -2.145796183 |
| Insc | -2.133412094 |
| 5830418P13Rik | -2.122269437 |
| Inpp5j | -2.104588743 |
| Scube2 | -2.100033315 |
| Fam167a | -2.096458511 |
| Gm40634 | -2.080218278 |
| Card11 | -2.068602686 |
| Fabp3 | -2.061418024 |
| Fhl1 | -2.059835284 |
| Gm10549 | -2.052804401 |
| Tecta | -2.050647033 |
| Col8a2 | -2.04863296 |
| Fzd10os | -2.027559167 |
| 5033430I15Rik | -1.994724651 |
| Cryab | -1.988232754 |
| Igsf1 | -1.978835619 |
| Stmn4 | -1.968791858 |
| Rbp7 | -1.944279059 |
| 2900009J06Rik | -1.940250991 |
| Atf3 | -1.927267958 |
| Odf3l1 | -1.92608959 |
| F2rl1 | -1.92195513 |
| Ndrg4 | -1.919536305 |
| Myh2 | -1.912727529 |
| Gm11756 | -1.887632805 |
| Gm7478 | -1.884149339 |
| Zp2 | -1.874802013 |
| Klhl34 | -1.858340491 |
| Npr3 | -1.842528364 |
| Ptprr | -1.83330235 |
| Mfap4 | -1.82075659 |
| Crnde | -1.816120256 |
| Dusp18 | -1.807287261 |
| Smtnl1 | -1.803624663 |
| Fabp3-ps1 | -1.802560164 |
| Themis3 | -1.799054698 |
| Arxes1 | -1.79845211 |
| Myl3 | -1.794457379 |
| Ppp1r14bl | -1.79409732 |
| Rbp4 | -1.784268924 |
| Gm45670 | -1.778878801 |
| Gm4804 | -1.775548608 |
| Myom3 | -1.773730422 |
| Mest | -1.745246625 |
| Col2a1 | -1.732563119 |
| Gm45867 | -1.726160208 |
| Dapp1 | -1.720255696 |
| Tceal7 | -1.71843068 |
| Atp1b4 | -1.662767813 |
| 1700027J07Rik | -1.642150364 |
| 1700056E22Rik | -1.641011146 |
| Fam171b | -1.637223848 |
| Mycn | -1.634646545 |
| Hspb7 | -1.626979391 |
| BC037032 | -1.6193672 |
| Ostn | -1.618854717 |
| Fhl2 | -1.617741516 |
| Apol6 | -1.606877179 |
| BC048679 | -1.591992352 |
| Slc7a10 | -1.572538773 |
| Tnnc1 | -1.572435776 |
| Dact2 | -1.561812922 |
| Myoz2 | -1.556198799 |
| Cuzd1 | -1.554064654 |
| Mettl21c | -1.54954175 |
| Lamc2 | -1.534563259 |
| Gm37435 | -1.531937425 |
| Homer2 | -1.52674428 |
| Gm41183 | -1.524945942 |
| Hist1h4n | -1.523977171 |
| Ssu2 | -1.523338716 |
| Lenep | -1.508840447 |
| 9530020I12Rik | -1.506388776 |
| Cacna2d2 | -1.502080823 |
| Gm45091 | -1.500044352 |
| Gm826 | -1.498042794 |
| Uchl1 | -1.493204586 |
| Gm35290 | -1.4839271 |
| Spon2 | -1.482168593 |
| Gm50221 | -1.469290876 |
| Thbs4 | -1.4655884 |
| 2310016D23Rik | -1.465310988 |
| Nat8l | -1.46414587 |
| 1110020A21Rik | -1.461675006 |
| Gm38832 | -1.456874907 |
| Myh10 | -1.453497808 |
| Tbxa2r | -1.451033095 |
| Tmem253 | -1.444495066 |
| Pdlim1 | -1.43816135 |
| Gm2174 | -1.436342088 |
| Nnat | -1.433929319 |
| A530016L24Rik | -1.432178662 |
| Gm11716 | -1.432088535 |
| Mfap5 | -1.430275064 |
| Gm32352 | -1.425830133 |
| Enah | -1.424675916 |
| Gm31251 | -1.422211702 |
| Gm45338 | -1.421029936 |
| H1f4 | -1.420170656 |
| Gm30794 | -1.418527423 |
| Aldh1b1 | -1.41441945 |
| Adprhl1 | -1.407091267 |
| Ccdc142os | -1.40650172 |
| Irx3os | -1.399325774 |
| Fam81a | -1.394500253 |
| Astn2 | -1.391691173 |
| Oxct1 | -1.387650638 |
| Gm28651 | -1.385554549 |
| Dnaja4 | -1.382180974 |
| Perm1 | -1.380844982 |
| Hpdl | -1.372529136 |
| Alox12 | -1.371914366 |
| Gm17546 | -1.371446578 |
| Serpinb1c | -1.368827795 |
| Lmcd1 | -1.36550035 |
| Tnfrsf12a | -1.361974648 |
| Gm11967 | -1.35984648 |
| Abhd18 | -1.358297063 |
| Tnnt1 | -1.357250945 |
| Lcat | -1.351503489 |
| Wnt5b | -1.34872935 |
| Cnga3 | -1.347225822 |
| Rpl7a | -1.345028324 |
| Nrep | -1.344432173 |
| Hspa1l | -1.340339573 |
| Rgcc | -1.338205241 |
| Zdhhc23 | -1.332401986 |
| Gm36210 | -1.330398136 |
| Inpp4b | -1.329874358 |
| Gm3888 | -1.328445898 |
| Ung | -1.327255149 |
| Islr2 | -1.327121135 |
| 4930511M06Rik | -1.325647756 |
| Actn2 | -1.32234396 |
| Esrrb | -1.320894752 |
| Rmi2 | -1.315992204 |
| Creb5 | -1.313684329 |
| Chrna2 | -1.312212962 |
| Pcp4l1 | -1.310536297 |
| Lrrn2 | -1.308891849 |
| Kcnh2 | -1.304957798 |
| Map1a | -1.295777893 |
| Gm50194 | -1.295555747 |
| Cabp1 | -1.292588715 |
| Zfp385b | -1.291643834 |
| St3gal5 | -1.285726731 |
| Xirp1 | -1.281981233 |
| Scel | -1.280636089 |
| Gm50321 | -1.278372847 |
| Gm12002 | -1.27772722 |
| 2310065F04Rik | -1.277441537 |
| Tekt2 | -1.272982553 |
| Fn1 | -1.271733301 |
| Gm29773 | -1.270295384 |
| 4921536K21Rik | -1.269697923 |
| Slc15a5 | -1.256451199 |
| Cep112 | -1.255892848 |
| Gm16118 | -1.252197053 |
| Gm13772 | -1.249626869 |
| Lynx1 | -1.238067457 |
| Gm5560 | -1.233145256 |
| Fzd10 | -1.229261701 |
| Gstm7 | -1.22877371 |
| D630024D03Rik | -1.225959202 |
| Adam12 | -1.225070947 |
| Colca2 | -1.222403004 |
| Gm45012 | -1.220906393 |
| Gm44949 | -1.21941875 |
| Ldhb | -1.214647691 |
| Gm15743 | -1.214529307 |
| Cilp | -1.211511792 |
| Idh2 | -1.206843704 |
| Tfrc | -1.206102398 |
| E330011O21Rik | -1.201746424 |
| Ifi44 | -1.201404851 |
| Gm5864 | -1.200475117 |
| Gm15833 | -1.199387802 |
| Grb14 | -1.199209723 |
| Gm7607 | -1.193266974 |
| Mettl21e | -1.180073124 |
| Col6a2 | -1.176125355 |
| Tmem30b | -1.161825892 |
| 2210039B01Rik | -1.161176198 |
| Acsm3 | -1.157465961 |
| Scn2a | -1.15499623 |
| Slc47a1 | -1.153313597 |
| Mlf1 | -1.145713009 |
| Adamts14 | -1.141980529 |
| Dkk3 | -1.135866722 |
| Gm30970 | -1.135196518 |
| Slc66a2 | -1.132313737 |
| Tex38 | -1.13153698 |
| Gm5860 | -1.130448171 |
| Gm32200 | -1.129486128 |
| Lgi1 | -1.129178679 |
| Opn3 | -1.122222605 |
| Gm40477 | -1.121381135 |
| Pamr1 | -1.119784185 |
| Hspb6 | -1.119662738 |
| Adamtsl5 | -1.114067959 |
| H3f3c | -1.112577039 |
| Esrrg | -1.111219081 |
| Fbxl2 | -1.109032321 |
| Cxcl11 | -1.107857012 |
| Prima1 | -1.106121575 |
| Tiam1 | -1.100812547 |
| Prph | -1.100011961 |
| Pdgfrl | -1.097336336 |
| Ybx2 | -1.095454236 |
| A330009N23Rik | -1.093273705 |
| Cacna1a | -1.092706436 |
| H2bc6 | -1.092570457 |
| Atp1b1 | -1.086085426 |
| Pde10a | -1.085167577 |
| Gm8712 | -1.082853331 |
| Vgll2 | -1.075700943 |
| Slc22a4 | -1.075615122 |
| Fgfbp1 | -1.063844098 |
| Itgbl1 | -1.062772166 |
| Mdh1 | -1.062062509 |
| Amd-ps1 | -1.05848769 |
| Rcan2 | -1.056811213 |
| Hsbp1l1 | -1.054278453 |
| Nckap5 | -1.052737845 |
| Mfsd4b3-ps | -1.047795442 |
| Gm44502 | -1.047216074 |
| Oaf | -1.045952514 |
| Serpine1 | -1.044490224 |
| Gm6576 | -1.037406854 |
| Gm49708 | -1.035747393 |
| Irf5 | -1.029283322 |
| Gm16062 | -1.028743975 |
| Sez6l2 | -1.024600584 |
| Myh7 | -1.023255342 |
| Cdo1 | -1.02257615 |
| Cdc42ep2 | -1.020412108 |
| H19 | -1.01937804 |
| Gm44616 | -1.017386236 |
| 9430091E24Rik | -1.017323062 |
| Mtfp1 | -1.014113927 |
| Aplp1 | -1.013414508 |
| Gipc3 | -1.009657407 |
| Cox7a1 | -1.00699121 |
| Cnnm4 | -1.006675814 |
| Arc | -1.005674624 |
| Efr3b | -1.005174232 |
| Gm5499 | -1.00503585 |
| Cerox1 | 1.000792923 |
| Piwil2 | 1.001561782 |
| Rpl15-ps6 | 1.001984678 |
| Jpt1 | 1.002168361 |
| Tgfbr1 | 1.00265771 |
| Oscp1 | 1.004656206 |
| Akip1 | 1.010210563 |
| Tlr5 | 1.011632856 |
| Pcdhgc3 | 1.011721509 |
| Pfkfb3 | 1.011909378 |
| Cldn19 | 1.014011491 |
| Dtd2 | 1.014486169 |
| Vars | 1.015195178 |
| Crocc | 1.016573002 |
| Gprc5b | 1.018619833 |
| Ppp1r9a | 1.019052105 |
| Nrbp2 | 1.019933495 |
| Setd4 | 1.020257859 |
| Ephb6 | 1.020445772 |
| Hacd4 | 1.021132549 |
| Flot1 | 1.02131906 |
| Il16 | 1.021859967 |
| Slc2a3 | 1.022179158 |
| Rps6ka5 | 1.026135438 |
| Tbx3os1 | 1.027237486 |
| Rnf125 | 1.028003168 |
| Ovgp1 | 1.028431047 |
| Ddit4 | 1.029326957 |
| Vegfd | 1.033049202 |
| Zcchc24 | 1.035005217 |
| Ccndbp1 | 1.036103478 |
| Mocos | 1.036197776 |
| BC023105 | 1.037680009 |
| Trp53inp1 | 1.038568382 |
| Rassf4 | 1.04072424 |
| Stbd1 | 1.040832411 |
| Mib1 | 1.043671596 |
| Rorc | 1.044655205 |
| Uri1 | 1.046414045 |
| Cnksr1 | 1.047297558 |
| Slc12a2 | 1.047532219 |
| Tmem237 | 1.048325915 |
| Acsl3 | 1.048549199 |
| Cfap36 | 1.048657698 |
| Cpd | 1.052181818 |
| Tspan17 | 1.053319786 |
| Ier3 | 1.05445367 |
| Ubb | 1.055859358 |
| Pdlim3 | 1.056677023 |
| Nploc4 | 1.059107712 |
| Slc43a1 | 1.06055575 |
| Gm7049 | 1.061147295 |
| Tep1 | 1.062708316 |
| Zdhhc18 | 1.064060705 |
| Tubg2 | 1.064958472 |
| Gm5854 | 1.065391415 |
| Kif5a | 1.065773062 |
| Gm7160 | 1.067973696 |
| Igfbp5 | 1.068734257 |
| Sik1 | 1.071163507 |
| Gm39469 | 1.071499446 |
| Gm49492 | 1.072312185 |
| Mthfs | 1.074141569 |
| Hes1 | 1.074279996 |
| Ksr1 | 1.0762548 |
| Sox4 | 1.078923542 |
| Eif4b | 1.079254472 |
| Myc | 1.079878162 |
| Spc24 | 1.081002925 |
| Plekha7 | 1.084717713 |
| Pcdhb17 | 1.086530647 |
| Kif19a | 1.088220466 |
| Ptger4 | 1.08959682 |
| Grina | 1.091727408 |
| Gm14388 | 1.092432484 |
| F830016B08Rik | 1.092712618 |
| Nras | 1.093747675 |
| Psmd4 | 1.0940368 |
| Vsig10l | 1.098289596 |
| 1700109H08Rik | 1.098457954 |
| Ubb-ps | 1.099992662 |
| Sox10 | 1.100377183 |
| 3830408C21Rik | 1.10257344 |
| Spata24 | 1.102653572 |
| 2900052L18Rik | 1.104537075 |
| Zdbf2 | 1.108540135 |
| Tbcd | 1.109403309 |
| Mfsd7a | 1.10943781 |
| Pcdhb14 | 1.109650807 |
| Slc15a4 | 1.109722407 |
| Gm26885 | 1.110468463 |
| Syne1 | 1.110606887 |
| Zfp37 | 1.112185896 |
| Galnt15 | 1.117255536 |
| Ppp1r14b | 1.1173363 |
| Tgfbr3 | 1.117497328 |
| Arhgef37 | 1.120724792 |
| Ppp1r3f | 1.120750297 |
| Gm15675 | 1.12111714 |
| Cbx7 | 1.12494768 |
| Odc1 | 1.125477872 |
| Gm5422 | 1.128216735 |
| Tpr | 1.129441542 |
| Zdhhc12 | 1.130203251 |
| Aplnr | 1.130563943 |
| Itgb4 | 1.132108806 |
| Gm45512 | 1.132212669 |
| Il12a | 1.133602936 |
| Kcne4 | 1.13497446 |
| Nol3 | 1.135733093 |
| Fam161a | 1.135904636 |
| 1700096K18Rik | 1.137424593 |
| Chrnb1 | 1.137630395 |
| Arrdc1 | 1.137672182 |
| Scn7a | 1.138791074 |
| Fam181b | 1.139732036 |
| Chac1 | 1.140227494 |
| A730011C13Rik | 1.140546419 |
| Klhl5 | 1.141739319 |
| Nt5dc1 | 1.142106839 |
| Dsn1 | 1.144039136 |
| Rhbdf2 | 1.144105478 |
| 2410006H16Rik | 1.14618278 |
| A530013C23Rik | 1.146885721 |
| Tmem79 | 1.147391647 |
| Ptpn2 | 1.148689862 |
| Pde4b | 1.152696122 |
| Gm45250 | 1.155151587 |
| Camk2n2 | 1.156196987 |
| Fst | 1.157898796 |
| Hoxd3os1 | 1.159387109 |
| Cdh15 | 1.160113038 |
| Hip1 | 1.161224786 |
| Lrba | 1.163606741 |
| Tbc1d31 | 1.164858453 |
| Stat5a | 1.166362586 |
| 4833418N02Rik | 1.166661502 |
| Cldn5 | 1.167730437 |
| Gm20632 | 1.167966958 |
| Gm47644 | 1.169101068 |
| Psmd2 | 1.169503562 |
| Gm15446 | 1.17132977 |
| Rb1 | 1.171502724 |
| 2310010J17Rik | 1.172874954 |
| Mal | 1.173853236 |
| Gm10263 | 1.17789292 |
| Myh14 | 1.178117368 |
| Armcx5 | 1.178806668 |
| Tbc1d1 | 1.180073595 |
| Ptx3 | 1.180808237 |
| Akap11 | 1.181296789 |
| 4921507P07Rik | 1.181950174 |
| Tec | 1.183258484 |
| Teddm2 | 1.183482513 |
| Cd63 | 1.185011102 |
| Adh1 | 1.194551515 |
| 5031425E22Rik | 1.195488291 |
| Tmem88b | 1.19890012 |
| Anxa7 | 1.20039292 |
| Ggt5 | 1.201148778 |
| 0610043K17Rik | 1.202777036 |
| Mfsd2b | 1.204323841 |
| Spag6 | 1.204759782 |
| Slc10a6 | 1.204958774 |
| Rpl30-ps8 | 1.210439882 |
| Eif4ebp1 | 1.211135947 |
| Syne4 | 1.212661837 |
| Bbc3 | 1.214864383 |
| Magef1 | 1.218411273 |
| Pcdhga9 | 1.218714314 |
| C030034I22Rik | 1.219636204 |
| Myo5a | 1.220198251 |
| Hid1 | 1.220872842 |
| C130036L24Rik | 1.224900963 |
| Hmgb1-ps8 | 1.230206925 |
| 2810454H06Rik | 1.230869765 |
| A930033H14Rik | 1.231131969 |
| B230206H07Rik | 1.233594428 |
| Castor2 | 1.233839848 |
| Fxyd7 | 1.236123635 |
| Zbtb9 | 1.238969906 |
| Rpl15-ps3 | 1.239071567 |
| Gm42984 | 1.239518875 |
| Gramd1b | 1.240224697 |
| Ankrd10 | 1.243259346 |
| Pllp | 1.247307369 |
| Epb41l4aos | 1.250998446 |
| Gm11266 | 1.263165684 |
| Gm4841 | 1.269127648 |
| Il1r1 | 1.270879973 |
| Gm45102 | 1.274823252 |
| Bbs12 | 1.278349743 |
| Snhg15 | 1.278664951 |
| Bmper | 1.279988532 |
| Lonrf3 | 1.281691074 |
| Paqr6 | 1.281936822 |
| Abca5 | 1.283297598 |
| Hdac4 | 1.283604753 |
| Rab13 | 1.285989261 |
| Rsph10b | 1.286192572 |
| Gm14634 | 1.289680607 |
| Ephb1 | 1.293644739 |
| Mc5r | 1.299753237 |
| Steap4 | 1.300370378 |
| C1qtnf4 | 1.3040403 |
| Cd300a | 1.314215867 |
| Gpr182 | 1.314883922 |
| Eif5a2 | 1.316904248 |
| Pou3f1 | 1.31843231 |
| Traf3ip3 | 1.318725658 |
| Aldoc | 1.319052527 |
| Slc16a9 | 1.327917454 |
| Septin3 | 1.332026212 |
| Gm37305 | 1.332355927 |
| Limd2 | 1.341693901 |
| Pgf | 1.345927748 |
| Rnasel | 1.350597698 |
| Gm28557 | 1.351767543 |
| Klf10 | 1.352786348 |
| Fam71e1 | 1.359335679 |
| H6pd | 1.367025922 |
| Hs6st2 | 1.373661646 |
| Ntsr2 | 1.376293999 |
| Slc15a3 | 1.376982328 |
| Gm46404 | 1.3793183 |
| Gm12543 | 1.383086615 |
| Xpa | 1.384356474 |
| Nap1l1 | 1.385510973 |
| Ccdc122 | 1.387520718 |
| Mbp | 1.389263162 |
| Gm30122 | 1.389669136 |
| 2810405F17Rik | 1.392547311 |
| C730034F03Rik | 1.393431373 |
| Pcdhga2 | 1.39409548 |
| E2f2 | 1.395197411 |
| 1500015A07Rik | 1.396253468 |
| Snrpert | 1.400164026 |
| Gpm6b | 1.405468601 |
| Cirbp | 1.40651865 |
| Gdnf | 1.408154085 |
| Tmem140 | 1.409409395 |
| 1700028E10Rik | 1.411947281 |
| Rbm3-ps | 1.412142478 |
| Kcnq5 | 1.414111953 |
| Gas2l3 | 1.41579015 |
| Mme | 1.415865943 |
| Ugt8a | 1.417973736 |
| Il10ra | 1.423417433 |
| Ifi30 | 1.432406992 |
| Phf2os1 | 1.451901499 |
| Klhl38 | 1.463696638 |
| Jun | 1.463831193 |
| Ect2l | 1.469338719 |
| Gm13091 | 1.469881823 |
| D730003I15Rik | 1.476734918 |
| Acsl6 | 1.479385965 |
| Gnb3 | 1.482958672 |
| Cldn1 | 1.485215273 |
| Neil2 | 1.486511155 |
| Bhlhb9 | 1.492382811 |
| Hook2 | 1.512140199 |
| Marveld2 | 1.515220916 |
| Col28a1 | 1.522458187 |
| Gm16124 | 1.526991233 |
| Abca1 | 1.536330206 |
| Zc3h6 | 1.538611148 |
| Ccdc136 | 1.541243226 |
| Ctps2 | 1.545235397 |
| Mt3 | 1.553284428 |
| 6720489N17Rik | 1.55570252 |
| Prx | 1.563691085 |
| Neurl3 | 1.565770015 |
| Hsd11b1 | 1.578810037 |
| Vldlr | 1.579278279 |
| Pcdhb5 | 1.584217939 |
| Hgs | 1.593374109 |
| Gm48583 | 1.595742091 |
| Tlr2 | 1.599617942 |
| C330018A13Rik | 1.603774526 |
| Eda2r | 1.60478496 |
| Nfasc | 1.613636208 |
| Glud-ps | 1.615393039 |
| Cfap100 | 1.619302616 |
| A930030B08Rik | 1.623645343 |
| Rbm3 | 1.624096626 |
| Gjc3 | 1.626558481 |
| Adm | 1.630033678 |
| Snhg10 | 1.632755924 |
| Gm47218 | 1.641404746 |
| Bub1b | 1.650277391 |
| Myo1a | 1.650324629 |
| Bcas1 | 1.652724003 |
| Mir486 | 1.658129432 |
| Lpin2 | 1.673542854 |
| Tmem176a | 1.675117595 |
| Gm3650 | 1.681702913 |
| Gm7327 | 1.681922487 |
| Gpx3 | 1.686288133 |
| Ngfr | 1.689299808 |
| Avpr1a | 1.694386068 |
| Gpr37l1 | 1.697410695 |
| Gm16794 | 1.698487517 |
| Usp44 | 1.703052791 |
| Gm5611 | 1.708783811 |
| Trim63 | 1.709099416 |
| Arrdc3 | 1.709487702 |
| Dennd2c | 1.710417082 |
| Tgif1 | 1.712034945 |
| Cd82 | 1.719391725 |
| Cd247 | 1.720208826 |
| Gm10156 | 1.726248407 |
| Cpne2 | 1.730670206 |
| Nudt10 | 1.733464661 |
| Runx1t1 | 1.733635471 |
| Mmp9 | 1.739094777 |
| Ctxn3 | 1.743873703 |
| Gm4708 | 1.746749614 |
| Gm47071 | 1.750385314 |
| Rtn4rl2 | 1.753581824 |
| Gm7694 | 1.76747619 |
| Cd300ld | 1.768159597 |
| Inhbb | 1.77489581 |
| 2510046G10Rik | 1.783937249 |
| Tekt1 | 1.788110313 |
| Rps2-ps3 | 1.792340452 |
| Lpcat2 | 1.794142595 |
| Gm19486 | 1.79842174 |
| Inmt | 1.809387649 |
| Kif1a | 1.809517336 |
| Tmem184a | 1.823919018 |
| Dgat2l6 | 1.831455719 |
| Gm17334 | 1.83898315 |
| Gm45716 | 1.839481296 |
| Gm28529 | 1.852203812 |
| Fbxo32 | 1.864234064 |
| Apod | 1.866309034 |
| Gm14029 | 1.898429998 |
| Rdh16f1 | 1.902432247 |
| D430013B06Rik | 1.915205722 |
| Ncmap | 1.923617327 |
| Gm830 | 1.961555055 |
| Ccn5 | 1.967016414 |
| Dusp15 | 1.967228064 |
| Ccdc192 | 1.979527878 |
| Ifitm1 | 1.992040777 |
| Gm48584 | 1.994961886 |
| Pim1 | 2.002545162 |
| Csf2rb2 | 2.013211956 |
| F830208F22Rik | 2.02015062 |
| Gm45218 | 2.023680853 |
| Gm15411 | 2.035109781 |
| Gm7538 | 2.036003589 |
| Gm42432 | 2.064491924 |
| Smco3 | 2.069223347 |
| Car1 | 2.07640583 |
| Gm47920 | 2.106010969 |
| Hpgd | 2.107411932 |
| C1s2 | 2.121329848 |
| Gm5914 | 2.140249003 |
| Gm10603 | 2.156692354 |
| Gm49027 | 2.172723613 |
| Nog | 2.205098671 |
| Gmfg | 2.210138028 |
| Tppp2 | 2.25940951 |
| Gm43300 | 2.268826811 |
| Igfn1 | 2.271983582 |
| C2cd4a | 2.279432559 |
| Gm43797 | 2.291826009 |
| Cd300ld5 | 2.332929679 |
| Chrna1 | 2.352939076 |
| Col19a1 | 2.361454605 |
| Tgif2 | 2.374052603 |
| Cxcl1 | 2.38666418 |
| Gm48593 | 2.391886032 |
| Cfap53 | 2.397989995 |
| Chrna9 | 2.400560702 |
| Spns3 | 2.440388372 |
| Klc3 | 2.49262292 |
| Junb | 2.495622202 |
| Tmem100 | 2.532222212 |
| Zbp1 | 2.56994478 |
| Slc28a1 | 2.571947664 |
| Gadd45a | 2.585460202 |
| Ms4a4a | 2.586529221 |
| Gm15943 | 2.595654213 |
| Ccr1 | 2.647383019 |
| Clec4n | 2.674515126 |
| Cyp1b1 | 2.684211295 |
| Cpxm1 | 2.729266562 |
| Gm42417 | 2.774560361 |
| Cd300ld4 | 2.782231608 |
| Gm19056 | 2.789672359 |
| Slfn4 | 2.796939744 |
| Gpr85 | 2.799814136 |
| Chil1 | 2.832771242 |
| Gm14221 | 2.841259856 |
| Selp | 2.904356747 |
| Ggt6 | 2.910545565 |
| Csf3r | 2.911060174 |
| Cdh1 | 2.912746833 |
| Etfbl | 2.946644691 |
| 1700093K21Rik | 2.980821222 |
| Gm19807 | 3.050373723 |
| 4930512H18Rik | 3.09330218 |
| Gm33148 | 3.138535262 |
| Cxcl10 | 3.187469802 |
| Gdf5 | 3.220589276 |
| Plekhh1 | 3.228099988 |
| Slfn1 | 3.257111179 |
| Mt2 | 3.365588594 |
| Gm45774 | 3.425026958 |
| Gm49673 | 3.445091879 |
| Aox2 | 3.476662088 |
| Chl1 | 3.485274529 |
| Shroom3 | 3.496058481 |
| Calr4 | 3.562493681 |
| Ebi3 | 3.65127205 |
| S100a8 | 3.666757416 |
| Lcn2 | 3.892817783 |
| Fetub | 3.907009596 |
| Nos1ap | 3.920910879 |
| Gm18856 | 3.922210532 |
| 4930579K19Rik | 4.06879469 |
| Rundc3a | 4.143545236 |
| Cstl1 | 4.159811161 |
| Poln | 4.407630382 |
| Serpina3n | 4.550487296 |
| Card14 | 4.622906902 |
| Saa2 | 4.760626132 |
| Chil3 | 4.842289894 |
| Gm45901 | 5.473680927 |
| Saa1 | 5.669461155 |
| C2cd4b | 5.883840467 |
| Cxcl13 | 6.079939034 |
| E130218I03Rik | 7.609414898 |
| H2ac18 | 9.757501389 |
| Cd7 | 11.7514813 |
| Gm45062 | 11.79584621 |
| Gm49391 | 11.82939637 |
| Gm45487 | 11.87690571 |
| Fpr2 | 12.12035345 |
| Cd300ld2 | 12.17083162 |
| Frs3os | 12.27376064 |
| Camp | 12.30668927 |
| Gm16174 | 12.59505385 |
| Gm36107 | 12.68452291 |
| Gm42878 | 12.98788099 |
| Gm50316 | 13.07831373 |
| Cxcl5 | 13.13282315 |
| Gm15657 | 14.13647238 |
| Mmp13 | 14.14782023 |
| Gm26476 | 14.33573647 |
| Gm20716 | 15.56555464 |
| Saa3 | 17.35382517 |
