# Supplementary material for: A non-invasive mouse model that recapitulates disuse-induced muscle atrophy in immobilized patients
Source: Sci Rep. 2023 Dec 14;13:22201. doi: 10.1038/s41598-023-49732-8 (PMC10721881; doi:10.1038/s41598-023-49732-8)
Supplement: Supplementary file 2 — Supplementary Information 2. [file 41598_2023_49732_MOESM2_ESM.docx]

**S**upplementary Table 1: TaqMan probe and primers for quantitative PCR

| Gene name | Assay ID |
| --- | --- |
| Csrp3 | Mm00443379_m1 |
| Ccn4 | Mm01200484_m1 |
| Tnfrsf11b | Mm00435451_m1 |
| Hdac4 | Mm01299553_m1 |
| Igfn1 | Mm00617360_m1 |
| Junb | Mm04243546_s1 |
| Cdh1 | Mm01247356_m1 |
| Nos1ap | Mm01290688_m1 |
| Hist2h2aa1 | Mm00501974_s1 |
| Tgfbr1 | Mm00436964_m1 |
| Fbxo32 | Mm00499523_m1 |
| Trim63 | Mm01185221_m1 |
| Mettl21e | Mm01256359_m1 |
| GAPDH | Mm99999915_g1 |
